# *In vivo* photoacoustic imaging of a nonfluorescent E2 crimson genetic reporter in mammalian tissues

**DOI:** 10.1117/1.JBO.25.4.046004

**Published:** 2020-04-20

**Authors:** Olumide Ogunlade, Cassandra Stowe, Amit Jathoul, Tammy Kalber, Mark F Lythgoe, Paul Beard, Martin Pule

**Affiliations:** a University College London, Department of Medical Physics and Biomedical Engineering, London, United Kingdom; b University College London, UCL Centre for Advanced Biomedical Imaging, Division of Medicine, London, United Kingdom; c University College London, UCL Cancer Institute, Research Department of Haematology, London, United Kingdom

**Keywords:** genetic reporters, photoacoustics, E2 crimson, fluorescent protein

## Abstract

**Significance:** Green-fluorescent protein (GFP)-like fluorescent proteins are used extensively as genetic reporters in fluorescence imaging due to their distinctive ability to form chromophores independent of external enzymes or cofactors. However, their use for photoacoustic (PA) imaging has not been demonstrated in mammalian tissues because they possess low PA signal generation efficiency in their native state. By engineering them to become nonfluorescent (NF), their PA generation efficiency was increased. This enabled the generation of *in vivo* contrast in mice, making it possible for GFP-like proteins to be used as PA genetic reporters in mammalian tissues.

**Aim:** The aim was to develop a darkened GFP-like protein reporter by modifying E2 crimson fluorescent protein (FP) in order to generate NF mutant proteins with high PA signal generation efficiency for *in vivo* imaging.

**Approach:** The absorbance, fluorescence, and PA amplitude spectra of purified protein solutions of the FP and engineered NF mutants were measured in order to identify the mutant with the highest PA signal generation efficiency. This mutant, referred to as NFA, and the native FP were then stably expressed in LS174T human colorectal tumor cells using a retroviral vector and tested for photostability under continuous pulsed illumination. To demonstrate the improvement in PA signal generation *in vivo*, cells expressing the FP and NFA mutant were injected subcutaneously in mice and imaged using a Fabry–Perot based PA scanner.

**Results:** The NF mutants of E2 crimson exhibited fluorescence that was 2 orders of magnitude lower than the FP and a higher PA signal generation efficiency; the NFA-generated PA signal was approximately three times higher than the FP. Tumor cells expressing the NFA mutant provided sufficient image contrast to be visualized *in vivo* against a background of strong vascular contrast, whereas the FP-expressing cells did not generate visible contrast.

**Conclusion:** A GFP-like protein has been demonstrated as a genetic reporter for PA imaging in mammalian tissue for the first time. This was achieved by a mutation, which darkened the FP and increased the PA signal generation efficiency. The approach taken suggests that GFP-like proteins could be a promising addition to the current cohort of genetic reporters available for *in vivo* PA imaging.

## 1 Introduction

Genetic reporters can provide valuable insight into disease processes at a cellular or molecular level.^1^ Previously demonstrated reporters for photoacoustic (PA) imaging are broadly based on the genetic encoding of an enzyme, which produces an absorbing chromophore or the direct genetic encoding of an absorbing protein.^2^ An example of the enzymatic approach is the genetic encoding of tyrosinase,^3^^–^^6^ which catalyzes cellular tyrosine to form the chromophore eumelanin, which exhibits strong absorption at visible and near-infrared wavelengths. Enzymatic reporters are advantageous because one molecule of an enzyme can catalyze the biosynthesis of several chromophores, thereby providing inherent signal amplification. However, the synthesis of multiple chromophores for every molecule of enzyme expressed may impose a burden on cellular resources and jeopardize normal cellular functions.^7^ Genetic reporters based on the direct encoding of absorbing proteins are less likely to impose such a cellular burden because they offer a 1:1 stoichiometric mapping between the level of reporter gene expression and the amount of chromophore produced. The two main classes of directly encoded protein-based reporters that have been explored for PA imaging are bacterial phytochrome-based proteins and green-fluorescent protein (GFP)-like proteins. The former include near-infrared fluorescent proteins, which have an absorption peak between 640 and 710 nm, making them advantageous for exploiting the near-infrared optical imaging window^8^^,^^9^ in order to achieve high penetration depth. However, the presence of fluorescent emission and ground-state depopulation can result in low PA signal amplitude.^2^ Furthermore, this class of reporter relies on the presence of biliverdin to generate contrast, which necessitates the administration of exogenous biliverdin if they are used to visualize noneukaryotic cells.^10^ A subset of bacterial phytochrome-based reporters are photoswitchable phytochrome proteins, in which the absorption spectrum of the chromophore can be modified by changing the illumination wavelength.^11^^,^^12^ By taking a pixelwise difference between PA images acquired before and after changing the wavelength, the background hemoglobin signal is suppressed. This enhances sensitivity, which is advantageous for increasing the signal-to-noise ratio (SNR) in deep tissue imaging applications. Disadvantages of photoswitchable proteins include an increase in total image acquisition time due to the acquisition of multiple images in addition to the photoswitching time as well as the additional experimental complexity of requiring two laser beams for PA signal generation and photoswitching.

GFP-like reporters originate from the GFP family. These can autocatalytically synthesize an absorbing protein enabling their expression in almost any type of aerobically living cell without the need for external co-factors.^13^^,^^14^ Chromoproteins are a subgroup of GFP-like proteins that exhibit negligible fluorescence in their natural state.^15^ Although this makes them well suited as PA genetic reporters in terms of providing high signal generation efficiency, they have not been engineered for use as genetic reporters to the same extent as their fluorescent protein (FP) counterparts. As a consequence, the *in vivo* PA imaging of chromoprotein expression in mammalian cells has not been demonstrated to date.^16^ An alternative approach that may offer a faster route to the *in vivo* deployment of GFP-like proteins is the use of existing FP variants as they have been extensively engineered for use in a wide variety of cell types. However, in their unmodified form, they can exhibit low photostability and reduced PA signal amplitude^17^ due to the presence of fluorescence and ground-state depopulation. As a consequence, their application has been limited to small and relatively translucent organisms, such as the zebrafish and fruit fly pupa.^18^^,^^19^ These limitations can potentially be overcome by mutating the protein sequence to quench its fluorescence and derive darkened mutants, which possess the essentially nonfluorescent (NF) property of chromoproteins and thus exhibit high PA generation efficiency and photostability. This is the approach adopted in this study.

E2 crimson FP was chosen as a suitable candidate for this purpose because it is derived from DsRed,^20^ which can be engineered to be NF by four mutations.^21^ Additionally, E2 crimson FP has one of the highest reported molar extinction coefficients ($126,000\;M^{-1}\;{\mathrm{cm}}^{-1}$) and redshifted peak absorption wavelengths (611 nm) of the GFP-like FPs,^20^^,^^22^ making it potentially suitable for *in vivo* PA imaging at longer wavelengths (>600  nm), which offer better penetration depth.^23^ In this study, a number of NF mutants of E2 crimson FP have been engineered and characterized in terms of their optical and PA properties. We demonstrate that the NF mutants have a higher PA signal generation efficiency compared to E2 crimson FP. In addition, we stably expressed an NF mutant in the LS174T human colorectal tumor cell line using a retroviral vector. Finally, the transduced cells were visualized *in vivo* after subcutaneous injection in mice, whereas cells transduced with E2 crimson FP could not be visualized. This preliminary study represents the first time a GFP homologue has been imaged *in vivo* in mammals using PA imaging.

## 2 Materials and Methods

### 2.1 Synthesis of Purified Proteins

To establish the ground truth optical and PA properties in the absence of confounding factors such as cellular scattering, purified protein solutions of the fluorescent E2 crimson FP and the engineered NF mutants were synthesized in the first instance. The procedure for engineering NF mutants from E2 crimson FP follows the method previously described.^17^ In brief, it involves a semirandom mutation of the amino acid Ser 146 in E2 crimson FP. This mutation resulted in four mutants (S146G, S146N, S146C, and S146A), which all had markedly reduced fluorescence compared to E2 crimson FP. The mutants are henceforth referred to as NFG, NFN, NFC, and NFA throughout this paper.

The bacterial expression vector pGex-6-p2 containing E2 crimson FP or mutants was introduced by heat shock into DH5—a transformation competent *E*scherichia *coli* strain C2523 (New England Biolabs). The use of expression vector pGex-6-p2 allows for the inducible expression of proteins under the control of the lac promoter as well as containing multiple cloning sites and the Glutathione *S*-transferase protein purification tag.^24^ After transformation, bacteria were expanded in 500 mL LB media containing $100\;\mu g\;{\mathrm{mL}}^{-1}$ carbenicillin in a bacterial incubator shaker at 37°C to an optical density ranging between 0.5 and 0.7 at 600 nm. Bacterial cultures were then supplemented with isopropyl b-D-1-thiogalactopyranoside (Fisher Scientific) to a final concentration of 1 mM for 3 h. The resultant bacteria were then pelleted and stored at −80°C until protein extraction, purification, and concentration. Protein extraction was done using the B-PER™ bacterial protein extraction reagent (Thermo Fisher Scientific) and purified using a Pierce™ GST spin purification kit (Thermo Fisher Scientific), as per manufacturer’s instructions. The proteins were then concentrated using Amicon™ Ultra 30K centrifugal devices, as per manufacturer’s instructions. The resulting concentration of each protein was measured using a nanodrop ND-1000 spectrophotometer before being normalized to $1\;\mathrm{mg}\;{\mathrm{mL}}^{-1}$.

After measurements of the absorbance, fluorescence, and PA spectra of the purified proteins, mutant NFA was determined to have the best PA characteristics and was therefore chosen for expression in an LS174T human tumor cell line using a retrovirus.

### 2.2 Transduction of Mammalian Cells to Express E2 Crimson FP and NFA Mutant

E2 crimson FP and NFA mutants were expressed in an LS174T human colorectal tumor cell line using a retrovirus. Both LS174T cells and HEK-293T packaging cells were grown in Iscove’s modified Dulbecco’s media (IMDM, “Lonza”) cell culture medium supplemented with 10% fetal calf serum (FCS, “Biosera”) and 1% glutamax (Invitrogen). To produce a retroviral supernatant, HEK-293T cells were plated in 100-mm tissue culture dish at a density of $2\times 10^{5}\text{ cells }{\mathrm{mL}}^{-1}$ for 24 h prior to transfection. Transfections were performed when cells were 50% to 70% confluent. A bulk transfection mixture was prepared, where 30  μL GeneJuice^®^ transfection reagent (Merck Millipore) was added to 470  μL of plain Roswell Park Memorial Institute Medium (RPMI-1640) for each supernatant to be produced. Following a 5-min incubation at room temperature, a total volume of 12.5  μg of DNA was added to each plate being transfected (for retroviral transfection: 3.125  μg RDF RD114 env plasmid, 4.6875  μg PeqPam-env gagpol plasmid, and 4.6875  μg SFG retroviral construct). Following the addition of plasmid DNA, the mixture was incubated for a further 15 min at room temperature prior to dropwise addition to the HEK-293T cell culture. Plates were gently agitated following transfection. Supernatant harvested at 48 h was stored at 4°C and was then combined with the 72 h harvest prior to aliquoting and storage at −80°C.

For transduction, $1\times 10^{6}$ LS174T cells were plated in a six-well plate and cultured overnight. Each well was transduced by replacing the medium with 2 mL of thawed retrovirus encoding the protein and polybrene transfection reagent (final concentration of $5\;\mu g\;{\mathrm{mL}}^{-1}$). After 48 h, cells were harvested using trypsin and were replated into T25 flasks with complete IMDM cell culture media and put in a 5% ${\mathrm{CO}}_{2}$ humidified incubator at 37°C to recover. When confluent, cells were moved to a T175 to allow sufficient numbers for sorting.

Fluorescence-activated cell sorting (FACS) was performed to sort cell populations, which were highly expressing E2 crimson FP or NFA mutant, using the coexpressed marker gene CD34. Cells were harvested and washed with phosphate-buffered saline (PBS), followed by staining with antihuman CD34 PE antibody clone 581, as per manufacturer’s instructions (Biolegend, Cambridge, UK). Following a 30-min staining incubation (at room temperature in the dark), cells were washed, resuspended in PBS + 10% FCS ($∼5\times 10^{6}\;\mathrm{cells}\;{\mathrm{mL}}^{-1}$), and placed on ice pending sorting using the BD FACS Aria™ fusion. After sorting, the cells were returned to the incubator and left to recover. A total of $5\times 10^{6}$ sorted cells expressing either E2 crimson FP or NFA were resuspended in 45  μL of PBS before being mixed with 45  μL of Matrigel™ prior to subcutaneous injection in the flank of nude mice.

### 2.3 *In-vitro* PA Characterization

A PA spectroscopic system^25^^,^^26^ was used to measure the PA amplitude spectra of samples of purified proteins and cells. Briefly, the system consists of a 30-Hz tunable fiber-coupled Nd:YAG pumped OPO laser (Spitlight 600, InnoLas Laser GmbH, Krailling, Germany) used to excite samples placed in a homemade cuvette. A $10\times 10\;{\mathrm{mm}}^{2}$ and 50  μm polyvinylidene fluoride film ultrasound sensor with a −3  dB bandwidth of 23 MHz was used to detect the generated PA signals. PA signals, generated at wavelengths between 500 and 650 nm in 5-nm steps, were averaged over 100 pulses before being recorded using a digitizer (NI PCI 5124). In addition to measuring the PA amplitude spectra of purified proteins and protein-expressing cells, the photostability of the cells for *in vivo* imaging was assessed by measuring the generated PA signal amplitude, as a function of the number of excitation pulses, for a continuous train of 20,000 pulses. A PA signal was recorded after every 100 pulses, the recorded signal representing an average of the 100 generated signals. The excitation wavelengths used were 610 nm for E2 crimson FP and 585 nm for NFA mutant, corresponding to the peak absorption wavelengths of each protein. The excitation fluence at the two wavelengths was 2.67 and $2.93\;\mathrm{mJ}\;{\mathrm{cm}}^{-2}$, respectively.

### 2.4 *In vivo* PA Imaging

**Fig. 1:**
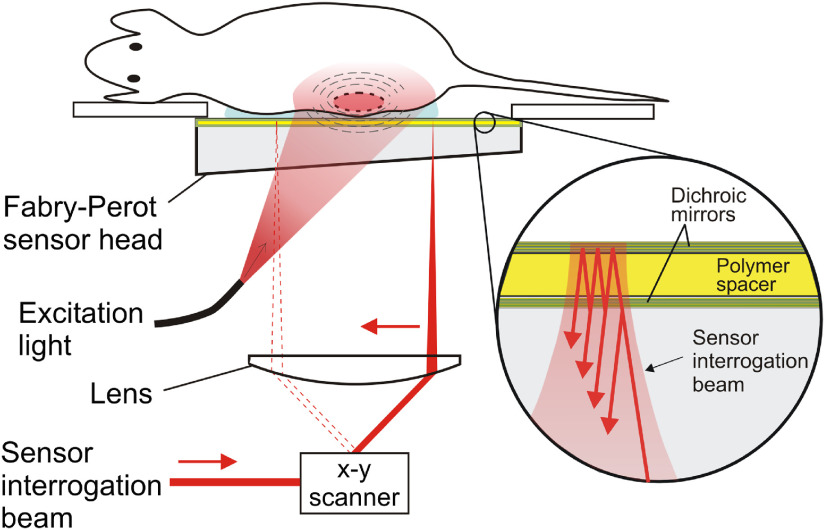
Schematic illustrating the operation of the planar FP PA scanner for *in vivo* imaging. Generated PA signals are detected by the FP polymer film ultrasound sensor. Inset: an expanded view of the sensor. It comprises a pair of dichroic mirrors separated by a polymer spacer thus forming a FP interferometer. The mirrors are transparent to the excitation laser wavelength between 585 and 620 nm used in this study but highly reflective to the sensor interrogation beam wavelength ∼1550  nm. The sensor is read out by raster scanning a focused continuous wave interrogation laser beam across it and measuring the change in the power of the reflected beam produced by acoustically induced changes in the spacer thickness.

*In vivo* PA signals were acquired using an all-optical, backward mode, planar PA scanner, which uses a Fabry–Perot (FP) polymer film interferometer that acts as an ultrasound sensor.^27^^,^^28^ Figure 1 shows a schematic of the operation of the scanner. Briefly, it consists of a fibre-coupled, tunable OPO laser system (Quanta Ray Pro-270/premiScan, Newport Spectra-Physics/GWU), which generates 7-ns excitation light pulses at wavelengths between 400 and 2000 nm. The divergent output of the optical fibre is transmitted through the sensor head, which is designed to be transparent to excitation wavelengths between 585 and 1200 nm; in this study, wavelengths in the range 585 to 620 nm were used. The diameter of the beam incident on the sensor was 2 cm and the fluence was $∼3\;\mathrm{mJ}\;{\mathrm{cm}}^{-2}$, which is below the maximum permitted exposure for skin.^29^ The absorption of the excitation light by the tissue generates PA waves, which propagate to the FP interferometer and modulates its optical thickness and thus its reflectivity. The latter is detected using a focused interrogation laser beam (around 1550 nm), tuned to the maximum slope of the interferometer transfer function, that is raster scanned over the sensor surface. The interrogation beam is reflected from the sensor and is incident on a photodiode, which is connected to a digitizer. Acoustic coupling between the sensor and the mouse is achieved using a small amount of water and an ultrasound gel applied to the surface of the mouse skin. Mice were anesthetized using isoflurane in oxygen [4% (vol/vol) at a flow rate of $2\;L\;\min^{-1}$ for induction and 1.5% (vol/vol) at a flow rate of $1\;L\;\min^{-1}$ for maintenance]. An integrated heater and thermal chamber maintained the body temperature of the anesthetized mice at 37°C. A typical scan over an area of $14\times 14\;{\mathrm{mm}}^{2}$, in steps of 100  μm, acquired ∼20,000 waveforms, each containing 500 time points with a temporal sampling interval of 20 ns, in about 7 min. All *in vivo* animal experiments were approved by a local ethical review panel at the University College London and were performed in accordance with the UK Home Office Animals Scientific Procedures Act (1986).

Three-dimensional (3D) PA images were reconstructed from the detected waveforms, using a time-reversal algorithm^30^ with a correction for acoustic attenuation in tissue using a time-variant filtering method.^31^ The image reconstruction algorithm was implemented using k-wave, an open-source MATLAB toolbox developed at University College London for the time-domain simulation and reconstruction of PA and ultrasound wave fields.^32^^,^^33^ Before reconstruction, the raw PA signals were interpolated onto a three times finer x–y grid. The sound speed used in the reconstruction was selected using an autofocus approach based on a metric of image sharpness.^34^ The reconstructed images were displayed as maximum intensity projections (MIPs) using a logarithmic image intensity scale or as 2D views of the volume-rendered 3D dataset. The volume rendering was implemented using Amira (FEI Visualization Sciences). To aid visualization, the cell population was manually segmented and false colored. For the segmentation, a volume of interest (VOI) within the reconstructed volume was selected, corresponding to the known location of the injected cells. The selected VOI was then displayed on a crimson color scale while the remaining volume was displayed in grayscale.

## 3 Results

**Fig. 2:**
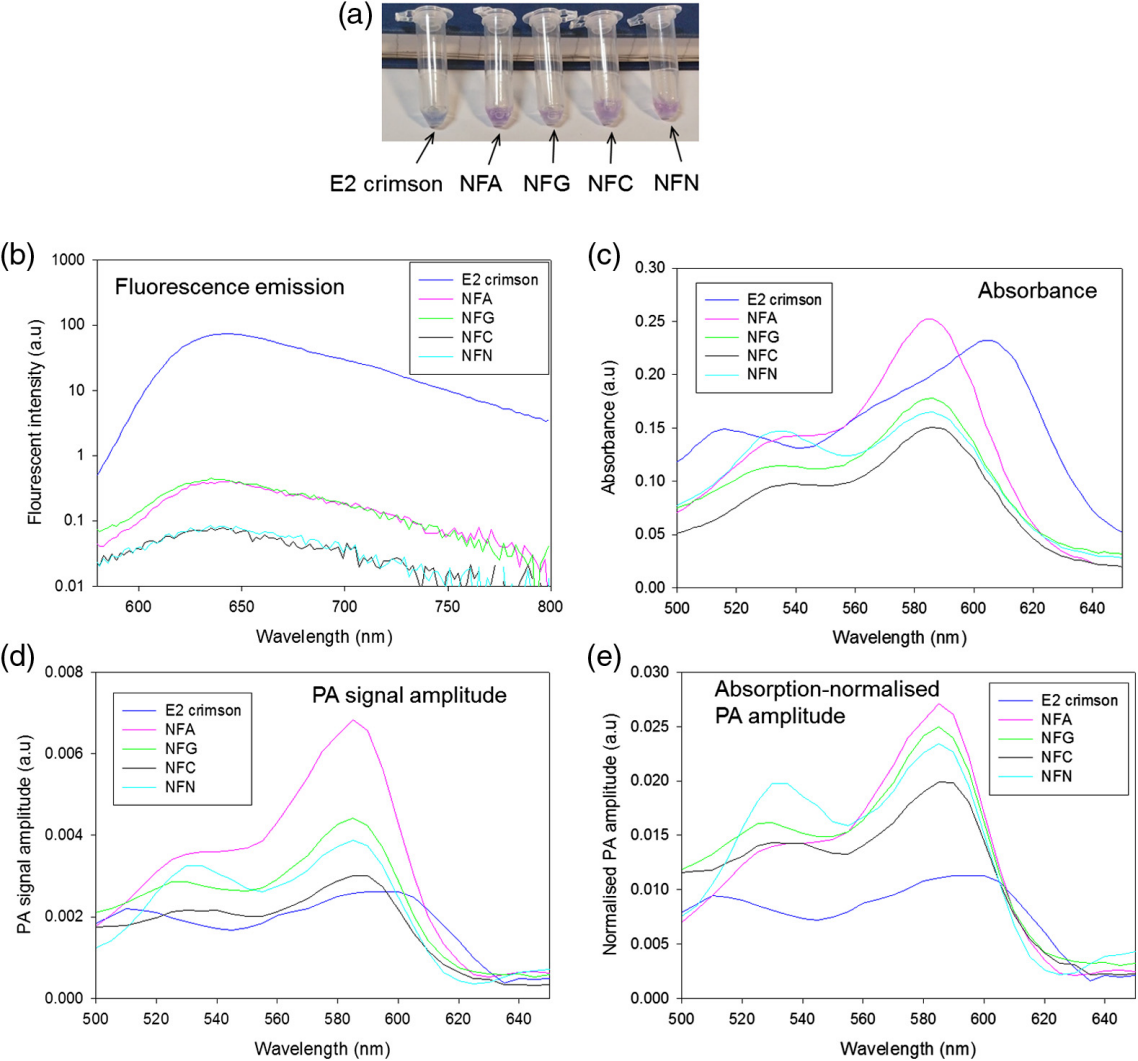
*In vitro* characterization of $1\;\mathrm{mg}\;{\mathrm{mL}}^{-1}$ solutions of purified proteins. (a) Photograph of purified proteins; the light blue color of the purified E2 crimson FP solution is in contrast to the light purple color of the NF mutants. (b) Fluorescence emission spectrum; the fluorescence of the NF mutants is at least 2 orders of magnitude lower than E2 crimson FP. (c) Absorbance spectrum; the absorbance peak of the NF mutants exhibits a small blue shift (585 nm) relative to E2 crimson FP (610 nm). (d) PA signal amplitude spectrum; all the NF mutants give higher PA signal amplitude than E2 crimson FP. The PA signal amplitude of mutant NFA is approximately three times higher than E2 crimson FP. (e) PA signal amplitude normalized by the peak absorption; the absorption-normalized PA signals from the NF mutants are approximately two to three times greater than the FP.

Purified protein solutions of E2 crimson FP and NF mutants were prepared for characterization, as described in Sec. 2. A photograph of the purified protein solutions [Fig. 2(a)] shows that the color of E2 crimson FP is different from the NF mutants. The fluorescent emission spectra of the solutions [Fig. 2(b)] show that the fluorescence intensity of E2 crimson FP is ∼2 orders of magnitude greater than any of the NF mutants. This demonstrates that the S146 mutations are effective in transforming the E2 crimson FP, which has a reported quantum yield (QY) of 0.23,^20^ so that it exhibits the essentially NF behavior of of chromoproteins, which have QYs in the region of 0.001.^15^

An unintended consequence of the mutation is a small blue shift of the absorption peak to 585 nm, compared to 610 nm for the E2 crimson FP [Fig. 2(c)]. The absorbance of the NF proteins is also slightly reduced compared to the E2 crimson FP, with the exception of the E2 crimson S146A (NFA) mutant, which has a slightly higher peak absorbance. Despite the reduced absorbance, all the NF mutants generate higher peak PA amplitudes [see Fig. 2(d)] than the FP. The higher PA signal generated by the NF mutants is seen more clearly by normalizing the PA spectra by the peak optical absorption of each protein [Fig. 2(e)]. The absorption-normalized PA signal amplitude of the NF mutants is approximately two to three times higher than the FP, yet based on the increased thermalization efficiency (1-QY) of the NF mutants alone the PA signal should only be a factor of 1.3 higher. This suggests that in addition to the increase in thermalization efficiency, the absence of ground state depopulation in the NF mutants also contributes to the higher PA signal generation.^17^ NFA generated the highest PA signal of all the proteins, approximately three times that of the E2 crimson FP and was therefore chosen for expression in mammalian cells for further *in vitro* and *in vivo* studies. The E2 crimson FP was also expressed in the same mammalian cell line for comparison.

**Fig. 3:**
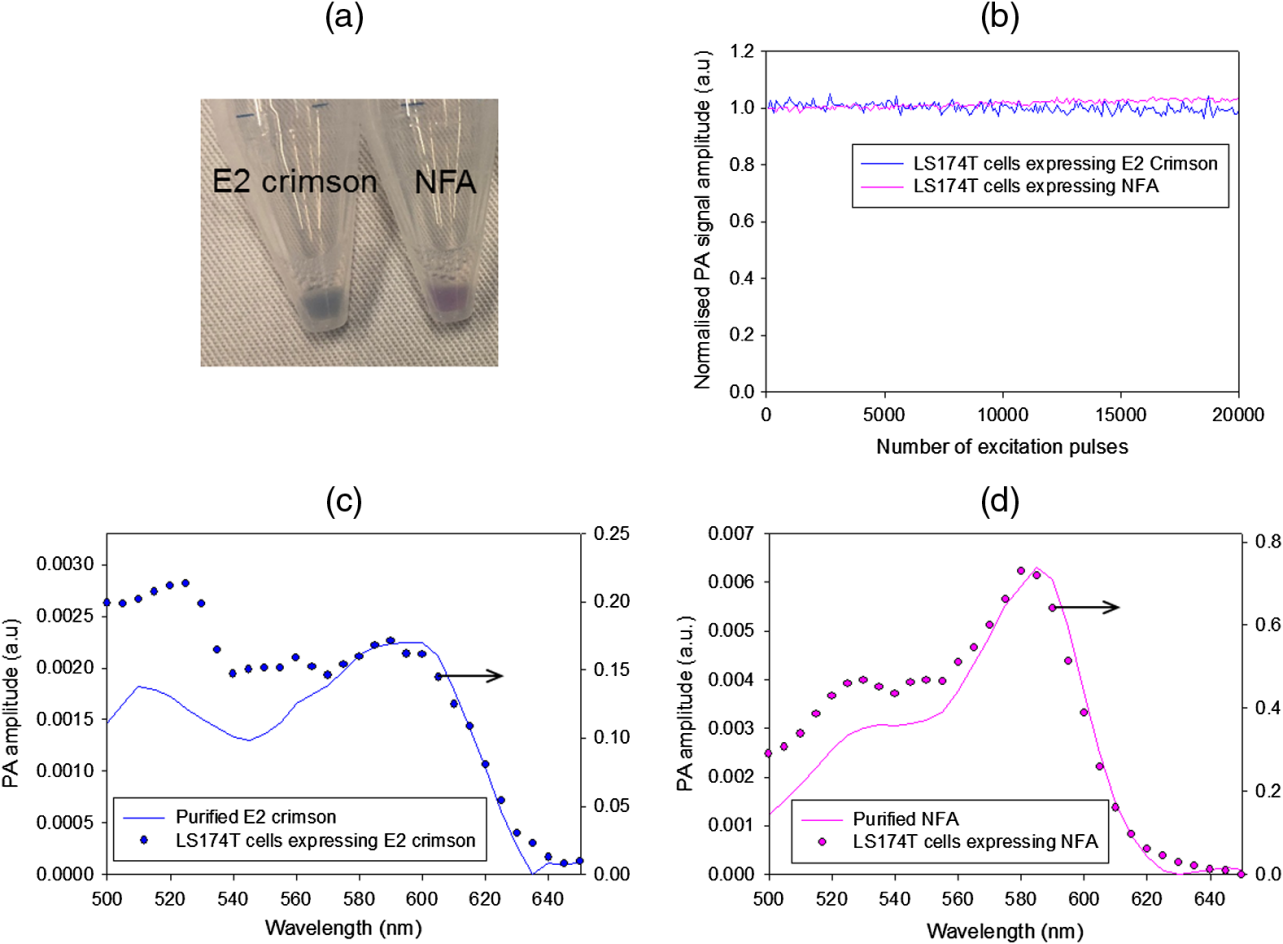
*In vitro* characterization of human colorectal tumor cells LS174T, expressing E2 crimson FP or NFA mutant. (a) Photograph of cell pellets after transduction. The contrast between the visual appearance of E2 crimson-expressing cells and the NFA mutant cells is similar to the purified proteins shown in Fig. 2(a). (b) Normalized PA signal amplitude from cells under prolonged exposure to nanosecond laser pulses. The excitation wavelength was 610 nm for the FP and 585 nm for NFA, corresponding to the peak absorption wavelengths of each protein. The incident fluence on the cells was 2.67 and $2.93\;\mathrm{mJ}\;{\mathrm{cm}}^{-2}$ respectively. There is no reduction in the PA signal amplitude, indicating high photostability. (c) PA amplitude spectra of cells compared to the PA spectra of the purified protein for (c) E2 crimson FP and (d) NFA mutant. The NFA mutant shows better agreement between the cells and the purified protein. The PA signal amplitude from the NFA cells is also greater than the FP.

After stable retroviral cell transduction in LS174T human colorectal tumor cells with vectors encoding E2 crimson and NFA mutant [Fig. 3(a)], the visible difference between the colors of the two cell populations is similar to that observed from the purified proteins in Fig. 2(a). We next exposed the cells to the high peak power PA excitation laser pulses at the peak absorption wavelength of the proteins. This was done in order to determine the photostability of the proteins at fluences typically used for *in-vivo* imaging. It has previously been shown that the PA signal amplitude generated by some GFP-like proteins decreases with the number of pulses.^17^ An assessment of the PA signal amplitude generated by the cells shows that it remains unchanged during exposure to 20,000 pulses at 30 Hz [Fig. 3(b)], thus demonstrating high photostability. The small increase in the PA amplitude from the NFA cells (3.6% at 20,000 pulses) was due to evaporation of PBS from the cuvette containing the cells during the experiment. In addition to assessing the photostability, the PA amplitude spectrum of the cells was also acquired. Figure 3(c) shows that the PA spectrum of the cells-expressing E2 crimson FP is in agreement with the PA spectrum of the purified protein for excitation wavelengths above 570 nm, although at shorter wavelengths, the spectra differ. In contrast, the NFA-expressing cells show good agreement across the entire spectrum [Fig. 3(d)], suggesting that the spectral properties of the protein are not significantly affected by its expression in mammalian cells.

**Fig. 4:**
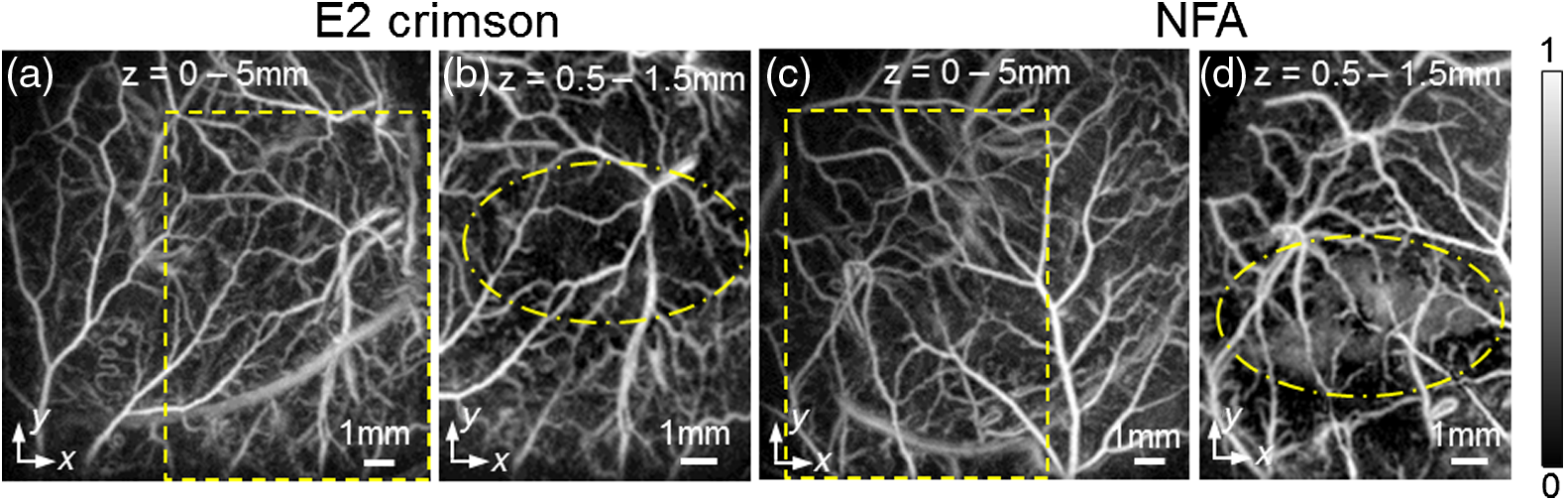
*In vivo* PA images of the flank of nude mice after subcutaneous injection of LS174T human colorectal tumor cells expressing E2 crimson FP or NFA mutant. The excitation wavelength was 600 nm. The images are horizontal x–y MIPs (a) MIP for z=0 to 5 mm after injection of E2 crimson-expressing cells. The dashed rectangle shows the region, where the cells are located (b) MIP for z=0.5 to 1.5 mm of the rectangular region from (a). The oval shape represents the boundary of where the cells are located. The E2 crimson-expressing cells are not visible. (c) MIP for z=0 to 5 mm after injection of cells expressing the NFA mutant. The dashed rectangle shows the region, where the cells are located (d) MIP for z=0.5 to 1.5 mm of the rectangular region from (c). The oval shape represents the boundary of the location of the NFA-expressing cells. The latter can be visualized within this region due to the generation of higher PA signals compared to the E2 crimson-expressing cells.

Figures 4(a)–4(d) show the PA images that were acquired after $5\times 10^{6}$ LS174T cells, expressing E2 crimson FP or NFA mutant, were injected subcutaneously into the flank of nude mice. The images are horizontal x–y MIPs acquired at an excitation wavelength of 600 nm. Figure 4(a) is an MIP for z=0 to 5 mm (0 mm represents the sensor surface), which shows that the E2 crimson FP cells are not visible after injection. In order to avoid the strong contrast from the superficial skin vasculature potentially obscuring weaker contrast from the cells, an MIP for depths greater than 0.5 mm was computed for the region highlighted by the dotted rectangle in Fig. 4(a). This is shown in Fig. 4(b) for z=0.5 to 1.5 mm. In this figure, the dotted ellipse represents the approximate boundary of the injection site of the cells. Even after excluding the contrast from skin vasculature in this way, the E2 crimson FP cells are still not visible [Fig. 4(b)]. Figure 4(c) shows the MIP for z=0 to 5 mm, which was obtained after injection of the NFA-expressing cells. The cells are not visible due to the high contrast from superficial vasculature. The highlighted rectangular region of Fig. 4(c) is shown in Fig. 4(d), as an MIP, for z=0.5 to 1.5 mm. Figure 4(d) shows that once the superficial vasculature has been removed, the NFA cells are visible within the dotted ellipse.

**Fig. 5:**
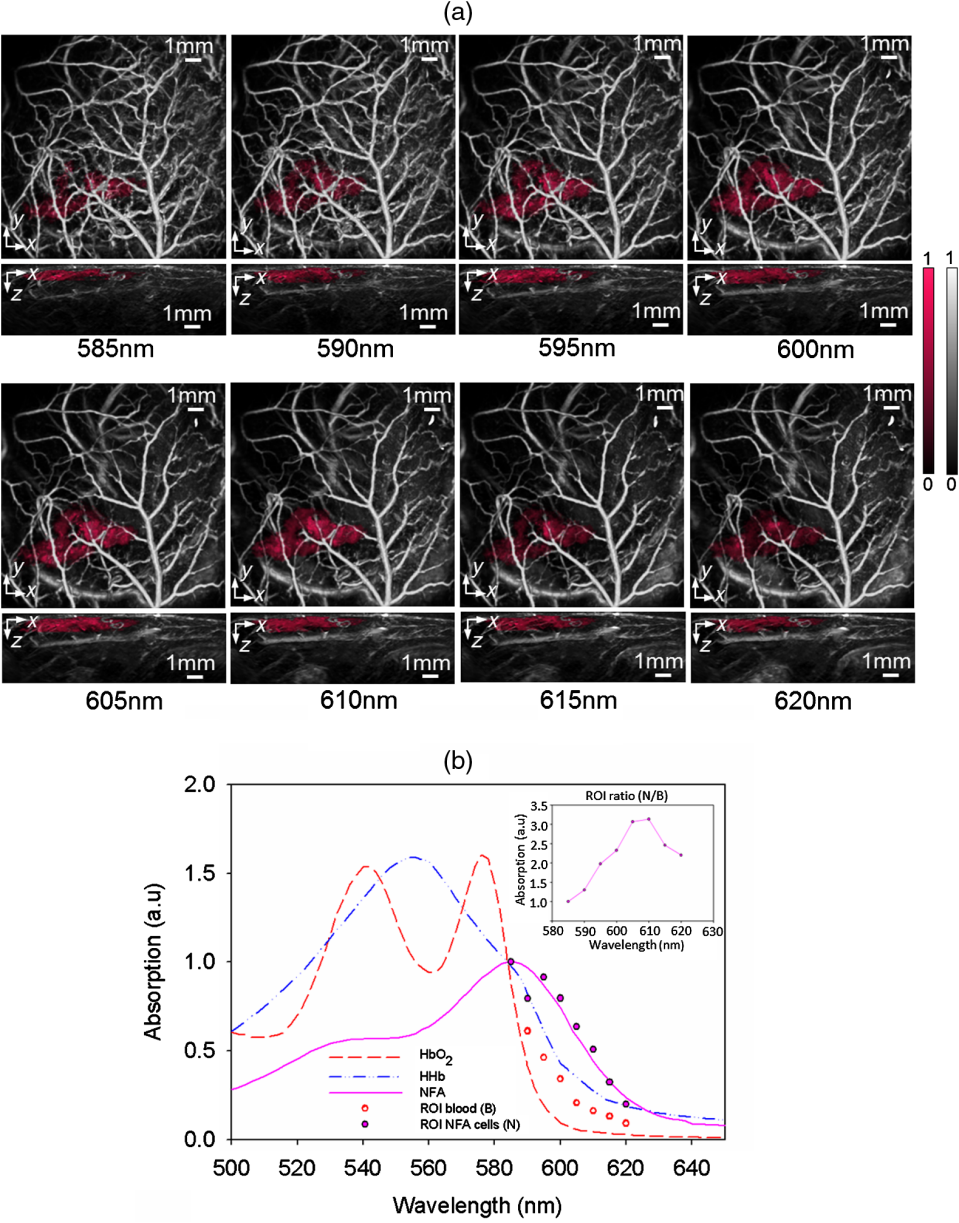
*In vivo* multiwavelength PA images acquired after subcutaneous injection of LS174T cells-expressing NFA mutant into the flank of a nude mouse. (a) Horizontal x–y and vertical x–z views of volume-rendered 3D datasets acquired at wavelengths between 585 and 620 nm. The size of the volume is $14\times 14\times 4.5\;{\mathrm{mm}}^{3}$. The cells have been manually segmented and false colored. (b) Normalized absorption of oxygenated (${\mathrm{HbO}}_{2}$), deoxygenated (HHb) hemoglobin, and purified NFA. For comparison, the wavelength-dependent mean intensity of ROIs from the *in vivo* PA images, which contain either the NFA cells (ROI NFA cells) or blood vessels (ROI blood) are also shown. The ROI spectrum of the NFA cells shows good qualitative agreement with the absorption spectrum of purified NFA. Inset: Ratio of the ROI spectrum of the NFA cells (N) to blood (B). The wavelength of maximum differential contrast for visualizing the cells is 610 nm.

Additional PA images were acquired at wavelengths between 585 and 620 nm in order to investigate the spectroscopic discrimination between the NFA cells and the surrounding vasculature; the lower limit of 585 nm is defined by the minimum excitation wavelength that the FP sensor is transparent to. The multiwavelength PA images acquired are shown in Fig. 5(a) as horizontal x–y and vertical x–z views of volume-rendered datasets, with the region of contrast provided by the NFA cells manually segmented and false colored. The images show the wavelength dependence of the differential PA contrast between the NFA cells and the surrounding vasculature. For example, at 600 nm, the cells are clearly visible. However, at the 585-nm peak absorption wavelength of the cells, the absorption of blood is much higher than at 600 nm (an order of magnitude higher for fully oxygenated blood), resulting in the cells appearing less visible. At 620 nm, the reduced absorption by blood results in higher contrast from the cells compared to the 585-nm image, despite the absorption of the cells being ∼1 order of magnitude lower at 620 nm.

To investigate the differential contrast in a quantitative manner, a region of interest (ROI) containing the NFA cells was manually selected on the PA image at 600 nm and used to create a mask. The mean PA signal intensity of pixels within the mask was then calculated. The same mask was used to obtain the mean PA signal intensity at other wavelengths. A plot of the mean PA signal against wavelength is shown in Fig. 5(b) (ROI NFA cells). For comparison, following a similar procedure, a plot of the mean PA signal due to the vasculature located near the cells is also shown (ROI blood), alongside the *in-vitro* absorption spectra of purified NFA and oxyhemoglobin and deoxyhemoglobin. The *in vivo* ROI spectrum of the NFA cells shows good qualitative agreement with the absorption spectrum of purified NFA. It is also distinct from the *in-vivo* ROI vascular spectrum, which lies between the absorption spectra of oxy-hemoglobin and deoxyhemoglobin. The ratio of the *in vivo* NFA signal to blood signal at each wavelength is plotted in the inset of Fig. 5(b). It shows that the wavelength of maximum differential contrast is 610 nm, even though the peak absorption wavelength of the cells is 585 nm. Wavelengths between 600 and 615 nm provide the highest *in-vivo* differential contrast by avoiding the high absorption of blood at the shorter wavelengths and the reduced absorption of the cells at the longer wavelengths.

## 4 Discussion and Conclusion

In this study, we engineered E2 crimson, a FP known to be expressed well in mammalian cells, in order to quench its fluorescence and increase its PA signal generation efficiency. Four mutants were engineered by a semirandom mutation of the amino acid S146 in E2 crimson. The fluorescence of each mutant was found to be over 2 orders of magnitude lower than E2 crimson FP while retaining at least 65% of its high optical absorption ($126,000\;M^{-1}\;{\mathrm{cm}}^{-1}$).^20^ All four mutants generated higher PA signal amplitudes than the FP. For example, the peak PA signal amplitude of mutant NFA was approximately three times higher. Although this higher PA signal includes a small contribution from the higher absorbance of the mutant (1.1 times higher than the FP), it is predominantly due to the absence of fluorescence. Fluorescence in the FP contributes to a reduction in PA signal by two distinct mechanisms. The first mechanism involves a reduction in the thermalization efficiency by a factor corresponding to the fluorescence QY due to the presence of radiative relaxation. The second mechanism results in a reduction in the effective absorption coefficient of the protein. This occurs because fluorescence results in longer excited state lifetimes (ns), which prevents the ground state from being sufficiently repopulated during the excitation laser pulse. The depopulation of the ground state reduces the probability of an incident photon being absorbed, which results in a reduction in the absorbed energy. The absence of ground state depopulation provides the greater contribution of the two mechanism by which the NF mutants generate a higher PA signal; for example, the absorption-normalized PA signal generated by NFA is approximately 2.5 times higher than the FP yet the thermalisation efficiency is only a factor of 1.3 higher.

When mutant NFA was stably expressed in an LS174T human colorectal tumor cell line and injected subcutaneously in mice, it gave visible *in vivo* contrast. On the other hand, the E2 crimson FP-expressing cells did not give detectable *in vivo* contrast because the FP generates lower PA signal amplitude compared to the NF mutant. By acquiring PA images at multiple wavelengths, the *in vivo* contrast from the NFA cells was observed to follow a similar wavelength dependence as the absorption of the purified protein, which is spectrally distinct from hemoglobin absorption. This, along with the high photostability of NFA [Fig. 3(b)], is conducive to the implementation of spectral unmixing methods.^35^ Although not required for the current study because the cells were superficially located and provided sufficient contrast, spectral unmixing can offer more sensitive discrimination, which may be required for low protein concentrations, although this can be challenging at large imaging depths when the SNR is low.^2^

The NF mutants in this study were engineered by a single-site mutation of the FP at the location of S146. However, the mutation also resulted in blue-shifting of the peak absorption wavelength of the mutants to 585 nm compared to 610 nm for the original FP. Additional mutations may be required to yield NF proteins, which retain the peak absorption wavelength of the FP or achieve a red-shift.^36^ For example, up to four site mutations were required to produce an NF derivative of DsRed, which had a slightly red-shifted absorption peak relative to the fluorescent version.^21^ Achieving a similar red-shifted NF mutant of E2 crimson could improve the *in-vivo* imaging contrast since the absorption of blood decreases by almost an order of magnitude between 585 and 610 nm.

In summary, this study represents the first *in vivo* demonstration of a GFP homologue as a PA reporter in mammalian tissue. This was achieved by engineering NF mutants of an FP, which is already extensively deployed as a mammalian cellular reporter in fluorescence imaging. This is an alternative approach to the optimization of a native NF chromoprotein for mammalian cellular expression. Our approach resulted in mutants of the FP, which exhibited the NF, photostable and high PA signal generation properties of chromoproteins while maintaining the ability of the FP to be expressed in mammalian cells. The preliminary *in vivo* results of this study suggest that with further development, GFP-like proteins could find practical application as PA genetic reporters, complementing the existing palette of PA reporters. Such applications could include the investigation of subtle biological behaviors, such as transcription and cellular signalling, where enzymatic reporters may be less suited due to the slow accumulation and clearance of any metabolized pigment.^17^ They could also find applications in the probing of cell types, which are incompatible with phytochrome reporters as well as in superficial imaging applications, where the background suppression capability of photoswitchable phytochromes for deep tissue imaging is not essential. A future prospect for extending the utility of GFP-like proteins to deep tissue PA imaging could lie in the development of photoswitchable GFP-like proteins.^37^
